# Supplementary material for: Interventions to improve linkage along the HIV-tuberculosis care cascades in low- and middle-income countries: A systematic review and meta-analysis
Source: PLoS One. 2022 May 12;17(5):e0267511. doi: 10.1371/journal.pone.0267511 (PMC9098064; doi:10.1371/journal.pone.0267511)
Supplement: S2 File — (PDF) [file pone.0267511.s002.pdf]

National Institute for Health Research

UNIVERSITY of York
Centre for Reviews and Dissemination

Systematic review

1. * Review title.

Give the working title of the review, for example the one used for obtaining funding. Ideally the title should state succinctly the interventions or exposures being reviewed and the associated health or social problems. Where appropriate, the title should use the PI(E)COS structure to contain information on the Participants, Intervention (or Exposure) and Comparison groups, the Outcomes to be measured and Study designs to be included.

Systematic review of interventions to improve linkage gaps along the TBHIV care cascade in low and middle-income countries

32 words remaining

2. Original language title.

For reviews in languages other than English, this field should be used to enter the title in the language of the review. This will be displayed together with the English language title.

50 words remaining

3. * Anticipated or actual start date.

Give the date when the systematic review commenced, or is expected to commence.

27/08/2018

4. * Anticipated completion date.

Give the date by which the review is expected to be completed.

31/12/2018

5. * Stage of review at time of this submission.

Indicate the stage of progress of the review by ticking the relevant Started and Completed boxes. Additional information may be added in the free text box provided.
Please note: Reviews that have progressed beyond the point of completing data extraction at the time of initial registration are not eligible for inclusion in PROSPERO. Should evidence of incorrect status and/or completion date being supplied at the time of submission come to light, the content of the PROSPERO record will be removed leaving only the title and named contact details and a statement that inaccuracies in the stage of the review date had been identified.
This field should be updated when any amendments are made to a published record and on completion and publication of the review.

The review has not yet started: No

Review stage	Started	Completed
Preliminary searches	Yes	Yes
Piloting of the study selection process	Yes	Yes
Formal screening of search results against eligibility criteria	Yes	No
Data extraction	No	No
Risk of bias (quality) assessment	No	No
Data analysis	No	No

Provide any other relevant information about the stage of the review here (e.g. Funded proposal, protocol not yet finalised).

6. * Named contact.

The named contact acts as the guarantor for the accuracy of the information presented in the register record.

Amrita Daftary

Email salutation (e.g. "Dr Smith" or "Joanne") for correspondence:

Dr. Daftary

7. * Named contact email.

Give the electronic mail address of the named contact.

amrita.daftary@mcgill.ca

8. Named contact address

Give the full postal address for the named contact.

5252 Boulevard de Maisonneuve
Montreal, QC H4A 3S5
Canada

9. Named contact phone number.

Give the telephone number for the named contact, including international dialling code.

14168322665

10. * Organisational affiliation of the review.

Full title of the organisational affiliations for this review and website address if available. This field may be completed as 'None' if the review is not affiliated to any organisation.

Research Institute of the McGill University Health Centre; World Health Organization

Organisation web address:

http://rimuhc.ca/

11. Review team members and their organisational affiliations.

Give the title, first name, last name and the organisational affiliations of each member of the review team. Affiliation refers to groups or organisations to which review team members belong.

Dr Amrita Daftary. Research Institute of the McGill University Health Centre
Dr Satvinder Singh. World Health Organization
Ms Stephanie Law. Research Institute of the McGill University Health Centre
Ms Angela Salomon. Research Institute of the McGill University Health Centre

12. * Funding sources/sponsors.

Give details of the individuals, organizations, groups or other legal entities who take responsibility for initiating, managing, sponsoring and/or financing the review. Include any unique identification numbers assigned to the review by the individuals or bodies listed.

Funded by World Health Organization (Department of HIV/Treatment and Care [TAC])

13. * Conflicts of interest.

List any conditions that could lead to actual or perceived undue influence on judgements concerning the main topic investigated in the review.

None

None known

14. Collaborators.

Give the name and affiliation of any individuals or organisations who are working on the review but who are not listed as review team members.

Dr Madhukar Pai. Research Institute of the McGill University Health Centre

15. * Review question.

State the question(s) to be addressed by the review, clearly and precisely. Review questions may be specific or broad. It may be appropriate to break very broad questions down into a series of related more specific questions. Questions may be framed or refined using PI(E)COS where relevant.

In people living with HIV or active TB, what interventions improve identification of active TB and HIV co-infection, respectively, and linkage to treatment for co-infection?

Specifically:
a. In people living with HIV, what interventions improve TB diagnosis and TB treatment initiation?
b. In people with active TB, what interventions improve HIV diagnosis and ART initiation?

193 words remaining

16. * Searches.

Give details of the sources to be searched, search dates (from and to), and any restrictions (e.g. language or publication period). The full search strategy is not required, but may be supplied as a link or attachment.

A primary search will be conducted using two databases: MEDLINE (using Ovid or PubMed), and EMBASE. We will also include original studies from the reference lists of both 1) published systematic reviews that relate to the TB and HIV diagnostic and treatment initiation care cascades (this includes reviews of TB screening and diagnostic interventions, HIV testing intervention, and TB-HIV service integration interventions) and 2) all articles ultimately included in our study. The search strategy will be developed in consultation with an expert librarian at McGill University, Canada, and implemented by the research team. It will combine terms, keywords and subject headings based on the following concepts: 1) tuberculosis; 2) HIV or AIDS; 2) screening / testing / diagnosis / case-detection / notification; 3) treatment initiation / prescription / commencement; 4) referral / linkage / integration / coordination / collaboration. Articles retrieved from the primary search will be combined in an Endnote file, and duplicates removed. The search strategy will be limited to studies published in English since 2003 (following the release of Stop TB'S "Guidelines for implementing collaborative TB/HIV programme activities"), and reporting primary data from LMICs.

113 words remaining

17. URL to search strategy.

Give a link to the search strategy or an example of a search strategy for a specific database if available (including the keywords that will be used in the search strategies).

Alternatively, upload your search strategy to CRD in pdf format. Please note that by doing so you are consenting to the file being made publicly accessible.

Do not make this file publicly available until the review is complete

18. * Condition or domain being studied.

Give a short description of the disease, condition or healthcare domain being studied. This could include health and wellbeing outcomes.

Tuberculosis (TB) and Human Immunodeficiency Virus (HIV) diagnosis and treatment initiation

189 words remaining

19. * Participants/population.

Give summary criteria for the participants or populations being studied by the review. The preferred format includes details of both inclusion and exclusion criteria.

Inclusions:
a. Persons living with HIV in care (newly diagnosed with HIV, with an existing HIV diagnosis, and/or receiving ART)
b. Persons with active TB or TB disease in care (newly diagnosed with active TB or TB disease, with an existing TB diagnosis, based on culture / smear, GeneXpert result, or clinical diagnosis)
c. Eligible persons of all ages (children (less than 18 y) and adults)

Exclusions: none

133 words remaining

20. * Intervention(s), exposure(s).

Give full and clear descriptions or definitions of the nature of the interventions or the exposures to be reviewed.

Include:
a. Any intervention that targets patients, providers or programs and impacts linkage to TB treatment and care among people living with HIV (i.e., diagnosis of active TB, entry into TB care, TB treatment initiation)
b. Any intervention that targets patients, providers or programs and impacts linkage to HIV treatment and care among people with active TB (i.e., diagnosis of HIV, entry into HIV care, ART initiation)

Exclude:
a. Interventions to prevent incidence of active TB among people living with HIV (e.g., LTBI screening and treatment)
b. Biomedical interventions (e.g., drug trial or drug test, diagnostic test or diagnostic tool)
c. Population level interventions (e.g., community awareness campaigns, general TB or HIV screening and testing interventions)

84 words remaining

21. * Comparator(s)/control.

Where relevant, give details of the alternatives against which the main subject/topic of the review will be compared (e.g. another intervention or a non-exposed control group). The preferred format includes details of both inclusion and exclusion criteria.

Local standard of care for TB/HIV

194 words remaining

22. * Types of study to be included.

Give details of the types of study (study designs) eligible for inclusion in the review. If there are no restrictions on the types of study design eligible for inclusion, or certain study types are excluded, this should be stated. The preferred format includes details of both inclusion and exclusion criteria.

We will include original studies (i.e., studies reporting primary data, cohort studies, randomized controlled trials (RCT) and quasi-randomized controlled trials).

We will exclude secondary analyses or reviews, commentaries, editorials, case-reports, studies with 10 participants, case-control studies, qualitative studies, unpublished studies, and studies published in non peer-reviewed journals.
100 words remaining

23. Context.

Give summary details of the setting and other relevant characteristics which help define the inclusion or exclusion criteria.

We will included only studies published in 2003 or later (following the release of Stop TB'S "Guidelines for implementing collaborative TB/HIV programme activities") and discussing interventions within low- and middle-income countries.
219 words remaining

24. * Main outcome(s).

Give the pre-specified main (most important) outcomes of the review, including details of how the outcome is defined and measured and when these measurement are made, if these are part of the review inclusion criteria.

There are four primary outcomes:
1. TB diagnosis (based on culture / smear / Xpert, or clinical diagnosis)
2. TB treatment initiation (any regimen used for ACTIVE TB; excludes preventative treatment (isoniazid))
3. HIV diagnosis (based on antigen / antibody / NAT test)
4. ART initiation (any regimen used for HIV treatment; excludes cotrimoxazole preventative therapy (CPT), Pre-Exposure Prophylaxis (PrEP), and TasP (Treatment as Prevention))
138 words remaining

Timing and effect measures

n/a
199 words remaining

25. * Additional outcome(s).

List the pre-specified additional outcomes of the review, with a similar level of detail to that required for main outcomes. Where there are no additional outcomes please state 'None' or 'Not applicable' as appropriate to the review

Secondary outcomes* include:
1. Diagnostic or treatment delay
2. Death

*Included studies must ALSO report on a primary outcome. Studies only reporting on a secondary outcome will be excluded.
273 words remaining

Timing and effect measures

n/a
299 words remaining

26. Data extraction (selection and coding).

Give the procedure for selecting studies for the review and extracting data, including the number of researchers involved and how discrepancies will be resolved. List the data to be extracted.

A data collection form will be developed and pilot-tested by the two independent reviewers, and refined or clarified as necessary. After finalizing each form, the two reviewers will use it to independently extract data from all eligible studies. If there are multiple reports of the same study, data from all reports will be extracted directly into a single data collection form. Any disagreement between reviewers will be resolved through discussion if possible. If the disagreement persists, it will be resolved in discussion with a third independent reviewer. When necessary, study authors will be contacted to obtain information required to resolve a disagreement. If a disagreement cannot be resolved after third-party consultation and after contacting study authors, it will be reported in the final review. All disagreements and their resolution will be clearly documented.
167 words remaining

27. * Risk of bias (quality) assessment.

State whether and how risk of bias will be assessed (including the number of researchers involved and how discrepancies will be resolved), how the quality of individual studies will be assessed, and whether and how this will influence the planned synthesis.

Two reviewers will independently appraise the quality of all included studies, on a distinct form, using the Cochrane risk of bias tool for RCTs, the ROBINS-I Tool for observational studies. The appropriateness of the statistical model used within studies will also be assessed, particularly relevant for quasi-experimental studies. The quality of each study will be classified as: very low, low, moderate or high. Any discrepancies between reviewers will be resolved in discussion or in consultation with a third reviewer. When necessary, study authors will be contacted to obtain missing information needed for quality assessment; in such cases, we will strive for open-ended questions in order to reduce the risk of overly positive responses.

87 words remaining

28. * Strategy for data synthesis.

Give the planned general approach to synthesis, e.g. whether aggregate or individual participant data will be used and whether a quantitative or narrative (descriptive) synthesis is planned. It is acceptable to state that a quantitative synthesis will be used if the included studies are sufficiently homogenous.

Given the anticipated diversity in types of interventions, summary effect measures will be estimated separately for each type of intervention (e.g. education and counselling; reminder and monitoring systems; financial incentives; etc.). For types of interventions and outcomes with sufficient number of studies, we will report pooled effect measures for those interventions (e.g. risk or rate ratios). The decision on whether meta-analyses should be conducted, and on which interventions and outcomes, will be arrived at through a discussion amongst the independent reviewers and research team. Considerations will include: number of studies; risk of biases of the potential studies to be included in a meta-analysis; methodological heterogeneity of the studies (participants, setting, study design, and modeling approach). In addition to any meta-analyses, we will report ranges of effect estimates for each type of intervention.

Meta-analyses will be done using a random effects model with R (V3.4.4).

155 words remaining

29. * Analysis of subgroups or subsets.

Give details of any plans for the separate presentation, exploration or analysis of different types of participants (e.g. by age, disease status, ethnicity, socioeconomic status, presence or absence or co-morbidities); different types of intervention (e.g. drug dose, presence or absence of particular components of intervention); different settings (e.g. country, acute or primary care sector, professional or family care); or different types of study (e.g. randomised or non-randomised).

We also plan to explore subgroup analyses for each of the following variables:
- study setting (e.g. by WHO region, rural/urban, income level,
- participant sex
- participant age group
- type of facility (e.g. clinic, hospital, community-based), and
- type of intervention (e.g. patient- or provider- or systems-focused, psychosocial or economic intervention)

Other potential subgroup analyses (contingent on range of data collected in included studies) include: baseline CD4 cell counts; severity of TB disease; site of TB (extrapulmonary or pulmonary); underlying TB or HIV drug resistance, previous TB and HIV treatment history; comorbidities (such as diabetes and mental illnesses, etc.), and specific population groups (such as prisoners, refugees, migrant workers, health care workers, injection drug users, etc.).

If appropriate, we will also conduct additional analyses to assess high- vs. low-quality studies, sensitivity analyses for each meta-analysis by removing each study one at a time to evaluate its impact on the pooled estimate, and publication bias using funnel plots and the Egger test.

92 words remaining

30. * Type and method of review.

Select the type of review and the review method from the lists below. Select the health area(s) of interest for your review.

Type of review

Cost effectiveness
No

Diagnostic
Yes

Epidemiologic
No

Individual patient data (IPD) meta-analysis
No

Intervention
Yes

Meta-analysis
Yes

Methodology
No

Narrative synthesis
No

Network meta-analysis
No

Pre-clinical
No

Prevention
No

Prognostic
No

Prospective meta-analysis (PMA)
No

Review of reviews
No

Service delivery
Yes

Synthesis of qualitative studies
No

Systematic review
Yes

Other
No

Health area of the review

Alcohol/substance misuse/abuse
No

Blood and immune system
No

Cancer
No

Cardiovascular
No

Care of the elderly
No

Child health

No

Complementary therapies
No

Crime and justice
No

Dental
No

Digestive system
No

Ear, nose and throat
No

Education
No

Endocrine and metabolic disorders
No

Eye disorders
No

General interest
No

Genetics
No

Health inequalities/health equity
No

Infections and infestations
Yes

International development
No

Mental health and behavioural conditions
No

Musculoskeletal
No

Neurological
No

Nursing
No

Obstetrics and gynaecology
No

Oral health
No

Palliative care
No

Perioperative care
No

Physiotherapy
No

Pregnancy and childbirth
No

Public health (including social determinants of health)
Yes

Rehabilitation
No

Respiratory disorders
No

Service delivery
Yes

Skin disorders
No

Social care
No

Surgery
No

Tropical Medicine
Yes

Urological
No

Wounds, injuries and accidents
No

Violence and abuse
No

31. Language.

Select each language individually to add it to the list below, use the bin icon to remove any added in error.
English

There is an English language summary.

32. Country.

Select the country in which the review is being carried out from the drop down list. For multi-national collaborations select all the countries involved.

Canada
Switzerland

33. Other registration details.

Give the name of any organisation where the systematic review title or protocol is registered (such as with The Campbell Collaboration, or The Joanna Briggs Institute) together with any unique identification number assigned. (N.B. Registration details for Cochrane protocols will be automatically entered). If extracted data will be stored and made available through a repository such as the Systematic Review Data Repository (SRDR), details and a link should be included here. If none, leave blank.
50 words remaining

34. Reference and/or URL for published protocol.

Give the citation and link for the published protocol, if there is one

Give the link to the published protocol.

Alternatively, upload your published protocol to CRD in pdf format. Please note that by doing so you are consenting to the file being made publicly accessible.

No I do not make this file publicly available until the review is complete

Please note that the information required in the PROSPERO registration form must be completed in full even if access to a protocol is given.

35. Dissemination plans.

Give brief details of plans for communicating essential messages from the review to the appropriate audiences.

Reporting of this systematic review and its meta-analyses will follow the PRISMA statement. We intend to publish the review upon completion.

Do you intend to publish the review on completion?

Yes

36. Keywords.

Give words or phrases that best describe the review. Separate keywords with a semicolon or new line. Keywords will help users find the review in the Register (the words do not appear in the public record but are included in searches). Be as specific and precise as possible. Avoid acronyms and abbreviations unless these are in wide use.

tuberculosis; human immunodeficiency virus; TB; HIV; linkage to care; coordination of care; diagnosis; treatment initiation

37. Details of any existing review of the same topic by the same authors.

Give details of earlier versions of the systematic review if an update of an existing review is being registered, including full bibliographic reference if possible.

50 words remaining

38. * Current review status.

Review status should be updated when the review is completed and when it is published.
Please provide anticipated publication date

Review_Ongoing

39. Any additional information.

Provide any other information the review team feel is relevant to the registration of the review.

40. Details of final report/publication(s).

This field should be left empty until details of the completed review are available.

Give the link to the published review.
