# Supplementary material for: Interventions to improve linkage along the HIV-tuberculosis care cascades in low- and middle-income countries: A systematic review and meta-analysis
Source: PLoS One. 2022 May 12;17(5):e0267511. doi: 10.1371/journal.pone.0267511 (PMC9098064; doi:10.1371/journal.pone.0267511)
Supplement: S4 File — (DOCX) [file pone.0267511.s004.docx]

## Supplemental 3: Quality assessments

### Table 1. Summary of quality assessment of non-randomized studies of interventions (NRSIs)

| **Ref** | **Study, year** | **Confounding Bias** | **Selection Bias** | **Classification bias** | **Bias due to deviations from interventions** | **Bias due to missing data** | **Outcome measurement bias ^z^** | **Reporting bias ^z^** | **OVERALL BIAS** |
| --- | --- | --- | --- | --- | --- | --- | --- | --- | --- |
| 1 | Agarwal, 2018 | Serious | Low | Low | Low | Low | Low | Low | Moderate |
| 2 | Ansa, 2014 | Serious | Low | Low | Low | Serious | Low | Low | Serious |
| 4 | Chukwuka, 2011 ^b^ | Not enough information | Low | Low | Low | Not enough information | Not enough information | Low | Not enough information |
| 5 | Courtenay-Quirk, 2018 | Serious | Low | Low | Low | Low | Low | Low | Serious |
| 6 | Herce, 2018 | Low | Low | Low | Low | Low | Low | Low | Low |
| 7 | Hermans S, 2012 | Serious | Low | Low | Serious | Moderate | Low | Low | Serious |
| 8 | Hermans SM, 2012 | Moderate | Low | Low | Low | Low | Low | Low | Moderate |
| 9 | Huerga, 2010 | Serious | Low | Low | Low | Serious | Low | Low | Serious |
| 10 | Ikeda, 2014 | Serious | Low | Low | Low | Low | Low | Low | Serious |
| 11 | Kanara, 2008 | Serious | Low | Low | Low | Low | Low | Low | Serious |
| 12 | Kaplan, 2016 | Low | Low | Low | Low | Moderate | Low | Low | Moderate |
| 13 | Kerschberger, 2012 | Low | Low | Low | Low | Low | Low | Low | Low |
| 15 | Louwagie, 2012 | Low | Moderate | Low | Low | Moderate | Low | Low | Serious |
| 16 | Mathebula, 2020 | Low | Low | Low | Low | Low | Low | Low | Low |
| 17 | Mwinga, 2008 | Serious | Low | Low | Low | Low | Low | Low | Serious |
| 18 | Nateniyom, 2008 | Moderate | Low | Low | Low | Moderate | Low | Low | Moderate |
| 19 | Ogarkov, 2016 | Serious | Low | Low | Low | Low | Low | Low | Serious |
| 20 | Owiti, 2015 | Moderate | Low | Low | Low | Moderate | Low | Low | Moderate |
| 21 | Rocha, 2011 | Serious | Low | Low | Moderate | Moderate | Low | Low | Serious |
| 22 | Van Rie, 2008 | Serious | Low | Low | Low | Moderate | Low | Low | Serious |
| 23 | Van Rie, 2014 | Moderate | Low | Low | Low | Low | Low | Low | Moderate |

1. Outcome measurement bias and Reporting bias were low for all studies, as outcomes were commonly abstracted from programmatic data and registers.
2. Study was an abstract and did not have enough information to assess quality, even after consulting with authors.

### Table 2. Summary of quality assessment of randomized controlled trials (RCTs)

| **Ref** | **Study, year** | **Random sequence generation** | **Allocation concealment** | **Blinding of participants and personnel** | **Blinding of outcome assessment** | **Incomplete outcome data** | **Selective outcome reporting** | **Recruitment bias** | **Baseline imbalance** | **Analysis bias** |
| --- | --- | --- | --- | --- | --- | --- | --- | --- | --- | --- |
| 3 | Auld, 2020 | Unclear | High | High | Low | Low | Low | Low | High | High |
| 14 | Kufa, 2018 | Low | N/A ^a^ | High ^b^ | Low | Low | Low | High | Low | High |

1. Allocation concealment considered not applicable in a cluster trial (as all clusters are usually randomized at once)
2. Little weight given to this domain, as blinding is impractical in cluster-randomized trial
