# Supplementary material for: Interventions to improve linkage along the HIV-tuberculosis care cascades in low- and middle-income countries: A systematic review and meta-analysis
Source: PLoS One. 2022 May 12;17(5):e0267511. doi: 10.1371/journal.pone.0267511 (PMC9098064; doi:10.1371/journal.pone.0267511)
Supplement: S5 File — (DOCX) [file pone.0267511.s005.docx]

## Supplemental 4: Grade evidence profiles

### PICO 1: HIV testing / ART initiation in people with active TB

**INTERVENTION 1: a. Co-located HIV Testing and TB services (Same Facility)**

| Certainty assessment | | | | | | | No of patients | | Effect | | Certainty | Importance |
| --- | --- | --- | --- | --- | --- | --- | --- | --- | --- | --- | --- | --- |
| **No of studies** | **Study Design** | **Risk of Bias** | **Inconsistency** | **Indirectness** | **Imprecision** | **Other considerations** | **Intervention** | **Control** | **Relative (95% CI)** | **Absolute (95% CI)** |  |  |
| HIV TESTING | | | | | | | | | | | | |
| 5^a^ | Observational ^b^ | Very Serious ^c^ | Serious ^d^ | Not Serious | Not serious | Most had several co-interventions ^e^ | 2430/3346 (72.6%) | 1173/2564 (45.5%) | 1.46 (1.39-1.53) | **209 more per 1,000** (from 177 more to 241 more) | ⊕🌕🌕🌕  Very Low | Critical |
| ART INITIATION | | | | | | | | | | | | |
| 3^f^ | Observational^g^ | Very Serious ^h^ | Serious ^i^ | Not Serious | Serious ^j^ | One study had several co-interventions ^k^ | 579/ 865 (66.9%) | 149/467 (31.9%) | 1.78 (1.37-2.19) | **249 more per 1,000** (from 118 more to 380 more) | ⊕🌕🌕🌕  Very Low | Critical |

1. Agarwal (2018) was conducted in standalone TB clinics in Ukraine from 2012-2015 and included all patients >18 years attending facilities offering inpatient/intensive treatment; Ansa (2014) was conducted in hospitals in Ghana from 2007-2008 and included all TB patients (new or previously diagnosed); Mwinga (2008) was conducted in hospitals and clinics in Zambia and included all TB patients registered at participating facilities; Nateniyom (2008) was conducted in TB clinics embedded within hospitals in Thailand and included all registered new diagnosed TB patients (excluding prisoners); Van Rie (2008) was conducted in TB clinics embedded within primary health facilities in DRC and included all TB patients >18 months registered at participating facilities.
2. Agarwal = non-randomized cluster-controlled before-and-after study; Ansa, Mwinga and Van Rie = Non-randomized, cluster-controlled study; Nateniyom = quasi-experimental (historical control)
3. Four studies have serious risk of bias due to confounding and missing data, the other has a moderate risk of bias due to confounding and missing data (no assessment or comparison of patient demographics).
4. RRs range from 1.26 (1.15-1.39) to 2.29 (2.03-2.57), many do not have overlapping 95 % CIs, and I^2^=94.1%, indicating significant heterogeneity
5. Systematic HIV testing (Agarwal, Mwinga, Nateniyom); other major operational improvements including an electronic data-management system & capacity building for providers (Agarwal).
6. Agarwal (2018) contributed two study arms (in TB and HIV clinics) in Ukraine from 2012-2015 and included all TB-HIV patients >18 years attending facilities offering inpatient/intensive treatment; Ansa (2014) was conducted in hospitals in Ghana from 2007-2008 and included all TB-HIV patients.
7. Agarwal = non-randomized cluster-controlled before-and-after study; Ansa = Non-randomized, cluster-controlled study
8. All studies have a serious risk of bias due to confound, and one has a serious risk of bias due to missing data.
9. RRs range from 1.49 (1.10-2.01) to 5.52 (2.68-11.38), 2 studies have non-overlapping 95% CIs and I^2^ = 79.2% indicating heterogeneity.
10. Wide confidence intervals in individual studies.
11. Major operational improvements including a new electronic data-management system & capacity building for providers (Agarwal)

**INTERVENTION 1 : b. Co-located HIV Testing and TB Services (Same Provider)**

| **Certainty assessment** | | | | | | | **No of patients** | | **Effect** | | **Certainty** | **Importance** |
| --- | --- | --- | --- | --- | --- | --- | --- | --- | --- | --- | --- | --- |
| **No of studies** | **Study Design** | **Risk of Bias** | **Inconsistency** | **Indirectness** | **Imprecision** | **Other considerations** | **Intervention** | **Control** | **Relative (95% CI)** | **Absolute (95% CI)** |  |  |
| HIV TESTING | | | | | | | | | | | | |
| 3^a^ | Observational^b^ | Very Serious^c^ | Serious ^d^ | Not Serious | Not Serious | None | 1763/2102 (83.9%) | 668/1794 (37.2%) | 1.47 (1.39-1.54) | **175 more per 1,000** (from 145 more to 201 more) | ⊕🌕🌕🌕  Very low | Critical |
| ART INITIATION | | | | | | | | | | | | |
| 1^e^ | Observational^f^ | Serious^g^ | Not Serious ^h^ | Not serious | Not serious | Study had co-interventions that are considered standard of care ^i^ | 118/196 (60.2%) | 192/600 (32.0%) | 0.53 (0.45-0.63) | **150 fewer per 1,000** (from 118 fewer to 176 fewer) | ⊕🌕🌕🌕  Very low | Critical |

1. Ansa (2014) was conducted in hospitals in Ghana from 2007-2008 and included all TB patients (new or previously diagnosed); Mwinga (2008) was conducted in hospitals and clinics in Zambia and included all TB patients registered at participating facilities; Van Rie (2008) was conducted in TB clinics embedded within primary health facilities in DRC and included all TB patients >18 months registered at participating facilities.
2. All three studies were non-randomized cluster-controlled trials.
3. All three studies have serious risk of bias due to confounding and missing data.
4. RRs range from 1.36 (1.26-1.47) to 1.43 (1.32-1.54) to 3.48 (3.12-3.89), do not all have overlapping 95% CIs, and I^2^=96.1%, indicating significant heterogeneity
5. Mwinga (2008) was conducted in hospitals and clinics in Zambia and included all TB patients newly diagnosed with HIV registered at participating facilities
6. Non-randomized cluster-controlled trial.
7. Serious risk of bias due to confounding; no comparison or adjustment of baseline imbalances.
8. Cannot assess inconsistency for a single study
9. Systematic HIV testing, healthcare worker training.

**INTERVENTION 1: c. Co-located TB and HIV Treatment (Same Facility)**

| Certainty assessment | | | | | | | No of patients | | Effect | | **Certainty** | **Importance** |
| --- | --- | --- | --- | --- | --- | --- | --- | --- | --- | --- | --- | --- |
| **No of studies** | **Study Design** | **Risk of Bias** | **Inconsistency** | **Indirectness** | **Imprecision** | **Other considerations** | **Intervention** | **Control** | **Relative (95% CI)** | **Absolute (95% CI)** |  |  |
| HIV TESTING | | | | | | | | | | | | |
| 0 |  |  |  |  |  |  |  |  |  |  |  |  |
| ART INITIATION | | | | | | | | | | | | |
| 1^a^ | Observational^b^ | Serious^c^ | Not Serious ^d^ | Not serious | Not serious | Both control and intervention arms had co-located testing (facility) | 74/105 (70.5%) | 104/233 (44.6%) | 1.58 (1.31-1.91) | **259 more per 1,000** (from 138 more to 406 more) | ⊕🌕🌕🌕  Very low | Critical |

1. Louwagie (2012); conducted in hospitals and primary health facilities in South Africa from 2011-2014; included all TB patients newly diagnosed with HIV.
2. Quasi-experimental (historical control)
3. Risk of bias due to missing data; as CD4 as not recorded for 68.7% of intervention group and 60.6% of control group, these participants were excluded from analysis as their eligibility could not be assessed.
4. Cannot assess inconsistency for one study

**INTERVENTION 1: d. Co-located TB and HIV Treatment (Same Provider)**

| Certainty assessment | | | | | | | No of patients | | | Effect | | **Certainty** | **Importance** |
| --- | --- | --- | --- | --- | --- | --- | --- | --- | --- | --- | --- | --- | --- |
| **No of studies** | **Study Design** | **Risk of Bias** | **Inconsistency** | **Indirectness** | **Imprecision** | **Other considerations** | **Intervention** | | **Control** | **Relative (95% CI)** | **Absolute (95% CI)** |  |  |
| HIV TESTING | | | | | | | | | | | | | |
| 0 |  |  |  |  |  |  |  | |  |  |  |  |  |
| ART INITIATION | | | | | | | | | | | | | |
| 1^a^ | Observational ^b^ | Serious^c^ | Not serious ^d^ | Not serious | Not serious | Both control and intervention arms had co-located testing (provider). Study had a co-intervention ^e^ | 354/513 (69.0%) | 62/373 (16.6%) | | 4.15 (3.28-5.25) | **523 more per 1,000** (from 378 more to 706 more) | ⊕🌕🌕🌕  Very low | Critical |

1. Van Rie (2014); conducted in primary health facilities in Democratic Republic of Congo from 2010-2012; included patients >18 years diagnosed with TB and HIV not yet initiated on ARTs
2. Quasi-experimental (historical control)
3. Authors note that age, gender, proportion smear negative and proportion with EPTB were similar between groups, but do not assess or control for any other potential confounders (CD4 count, other co-morbidities, socioeconomic status).
4. Cannot assess inconsistency for one study
5. Task-shifting of CD4-stratified ART initiation from clinicians to TB nurses

**INTERVENTION 1**: **e.** **Co-located HIV Testing plus TB and HIV Treatment (Same Facility)**

| Certainty assessment | | | | | | | No of patients | | Effect | | **Certainty** | **Importance** |
| --- | --- | --- | --- | --- | --- | --- | --- | --- | --- | --- | --- | --- |
| **No of studies** | **Study Design** | **Risk of Bias** | **Inconsistency** | **Indirectness** | **Imprecision** | **Other considerations** | **Intervention** | **Control** | **Relative (95% CI)** | **Absolute (95% CI)** |  |  |
| HIV TESTING | | | | | | | | | | | | |
| 0 |  |  |  |  |  |  |  |  |  |  |  |  |
| ART INITIATION | | | | | | | | | | | | |
| 2^a^ | Observational ^b^ | Serious ^c^ | Serious ^d^ | Not serious | Serious ^e^ | Both studies had several co-interventions ^f^ | 170/250 (68.0%) | 204/656 (31.1%) | 1.50 (1.07-1.93) | **156 more per 1,000** (from 22 more to 289 more) | ⊕🌕🌕🌕  Very low | Critical |

1. Owiti (2015) a study in hospitals and primary health facilities (PHFs) in Kenya between 2010 and 2012; Huerga (2010) a study in Kenyan TB clinic housed in a hospital between 2005 and 2007. Both studies included all newly registered TB/HIV co-infected patients.
2. Owiti (2015) is a non-randomized cluster-controlled trial comparing different levels of co-location to the standard-of-care (based on a referral system between separate TB and HIV clinics); Huerga (2010) is a pre/post analysis comparing the intervention to a historical control period.
3. Owiti (2015) and Huerga (2010) have moderate and severe risk of bias due to confounding and missingness, respectively.
4. Both studies conducted in public hospital/clinic settings in Kenya, however Owiti (2015) included many operational improvements and provider training, which were not specified by Huerga (2010).
5. The confidence interval for Owiti (2015) crosses the null and the intervention arm has a small sample size (n=39); imprecision was not a serious limitation in Huerga (2010).
6. Provider education and counselling (Huerga & Owiti), dedicated personnel (Huerga & Owiti), healthcare worker training (Owiti)

**INTERVENTION 1**: **f.**  **Co-located HIV Testing (Same Provider) plus TB and HIV treatment (Same Facility)**

| Certainty assessment | | | | | | | No of patients | | Effect | | **Certainty** | **Importance** |
| --- | --- | --- | --- | --- | --- | --- | --- | --- | --- | --- | --- | --- |
| **No of studies** | **Study Design** | **Risk of Bias** | **Inconsistency** | **Indirectness** | **Imprecision** | **Other considerations** | **Intervention** | **Control** | **Relative (95% CI)** | **Absolute (95% CI)** |  |  |
| HIV TESTING | | | | | | | | | | | | |
| 0 |  |  |  |  |  |  |  |  |  |  |  |  |
| ART INITIATION | | | | | | | | | | | | |
| 1^a^ | Observational ^b^ | Serious ^c^ | Not serious ^d^ | Not serious | Very Serious ^e^ | Both control and intervention arms had co-located testing (facility).  Study had co-interventions, some considered standard of care ^f^ | 112/155 (72.2%) | 6/99 (6.0%) | 11.92 (5.46-26.05) | **655 more per 1,000** (from 268 more to 1000 more) | ⊕🌕🌕🌕  Very low | Critical |

1. Ikeda (2014) a study in a Guatemala hospital from 2005-2006.
2. Ikeda (2014) a pre/post analysis comparing intervention to a historical control period in newly diagnosed HIV/TB co-infected patients aged over 15.
3. Ikeda (2014) has a serious risk of bias due to confounding
4. Cannot assess inconsistency for one study
5. Extremely wide confidence interval of (5.46-26.05)
6. Systematic HIV testing and extensive HCW training in HIV/TB co-infection (40% of providers received additional training in HIV integrated care through national 8-month diploma program)

**INTERVENTION 1**: **g.** **Co-located HIV Testing (Same Facility) plus TB and HIV Treatment (Same Provider)**

| Certainty assessment | | | | | | | No of patients | | Effect | | Certainty | Importance |
| --- | --- | --- | --- | --- | --- | --- | --- | --- | --- | --- | --- | --- |
| **No of studies** | **Study Design** | **Risk of Bias** | **Inconsistency** | **Indirectness** | **Imprecision** | **Other considerations** | **Intervention** | **Control** | **Relative (95% CI)** | **Absolute (95% CI)** |  |  |
| HIV TESTING | | | | | | | | | | | | |
| ^0^ |  |  |  |  |  |  |  |  |  |  |  |  |
| ART INITIATION | | | | | | | | | | | | |
| 2^a^ | Observational ^b^ | Serious ^c^ | Serious ^d^ | Not serious | Serious ^e^ | Both studies had several co-interventions ^f^ | 207/346 (59.8%) | 339/701 (48.5%) | 0.98 (0.87-1.10) | **10 fewer per 1,000** (from 63 fewer to 49 more) | ⊕🌕🌕🌕  Very low | Critical |

1. Owiti (2015) a study in hospitals and primary health facilities (PHFs) in Kenya between 2010 and 2012; Hermans SM (2012) a study in an HIV clinic in Uganda.
2. Owiti (2015) is a non-randomized cluster-controlled trial comparing different levels of co-location to the standard-of-care (based on a referral system between separate TB and HIV clinics); Hermans SM (2012) is a pre/post analysis comparing the intervention period to a historical control period. Both studies included all newly diagnosed HIV/TB co-infected patients.
3. Owiti (2015) and Hermans SM (2012) have moderate risk of bias due to confounding, and Owiti (2015) additionally has a moderate risk of bias due to missingness.
4. Hermans SM (2012) was conducted in an HIV clinic in Uganda whereas Owiti (2015) in a TB clinic in Kenya; similarly broad interventions with diverse operational improvements in addition to co-location; I^2^= 96.2%.
5. Hermans SM (2012) estimated confidence interval crosses the null; Owiti (2015) estimated a statistically significant effect but with a small sample size in the intervention arm (n=117).
6. Healthcare worker training (both), dedicated personnel (both), peer support (Hermans SM), patient education and counselling (Owiti) and major operational improvements including discussion of “difficult cases” at weekly team meetings, placement of ART initiation guides in clinic files, and phone-tracing to prevent loss to follow-up (Owiti)

**INTERVENTION 1**: **h.** **Co-located HIV Testing plus TB and HIV Treatment (Same Provider)**

| Certainty assessment | | | | | | | No of patients | | Effect | | **Certainty** | **Importance** |
| --- | --- | --- | --- | --- | --- | --- | --- | --- | --- | --- | --- | --- |
| **No of studies** | **Study Design** | **Risk of Bias** | **Inconsistency** | **Indirectness** | **Imprecision** | **Other considerations** | **Intervention** | **Control** | **Relative (95% CI)** | **Absolute (95% CI)** |  |  |
| HIV TESTING | | | | | | | | | | | | |
| 0 |  |  |  |  |  |  |  |  |  |  |  |  |
| ART Initiation | | | | | | | | | | | | |
| 4^a^ | Observational ^b^ | Not serious | Serious ^c^ | Not serious | Not serious | On study had co-located testing (facility) in both control and intervention arms ^d^  All studies had several co-interventions ^e^ | 280/480 (58.3%) | 355/806 (44%) | 1.45 (1.27-1.62) | **198 more per 1,000** (from 119 more to 273 more) | ⊕⊕  Low | Critical |

1. Owiti (2015) a study in hospitals and primary health facilities (PHFs) in Kenya between 2010 and 2012; Herce (2018) a study in two TB clinics in Zambia from 2010-2012; Kerschberger (2012) a study in a South African primary health facility from 2008-2009.
2. Owiti (2015) is a non-randomized cluster-controlled trial comparing different levels of co-location to the standard-of-care (based on a referral system between separate TB and HIV clinics); Herce (2018) and Kerschberger (2012) are pre/post analyses comparing the intervention period to a historical control period. All studies included all newly diagnosed HIV/TB co-infected patients.
3. Studies were conducted in diverse settings: Kerschberger (2012) in South African PHFs; Herce (2018) in Zambian PHF, and Owiti (2015) in Kenyan PHF/Hospitals. All included broad interventions that included diverse operational improvements in addition to co-location. However, not very serious because I^2^ = 0.0%
4. Kerschberger (2012).
5. Healthcare worker training (all), dedicated personnel (all), patient education and counselling (Herce and Owiti), and major operational improvements including dedicated ART clinic days, synchronized TB and HIV patient follow-up, combined health information system, patient filing system (with medical notes, screening tools, prescription charts) and monitoring/evaluation (Herce & Kerschberger).

**INTERVENTION 2**: **Patient education & counselling**

| **Certainty assessment** | | | | | | | **Impact** | **Certainty** | **Importance** |
| --- | --- | --- | --- | --- | --- | --- | --- | --- | --- |
| **No of studies** | **Study Design** | **Risk of Bias** | **Inconsistency** | **Indirectness** | **Imprecision** | **Other considerations** |  |  |  |
| HIV TESTING | | | | | | | | | |
| 1^a^ | Observational ^b^ | Serious ^c^ | Not serious ^d^ | Not serious | Not serious | Study had co-interventions ^e^ | HIV testing increased under the intervention: 96.9% of 318 TB patients tested for HIV under the intervention compared to 30.6% of 72 participants in the comparator group, RR 3.17 (2.24, 4.49). | ⊕🌕🌕🌕  Very low | Critical |
| ART INITIATION | | | | | | | | | |
| 2^f^ | Observational ^b^ | Serious ^g^ | Serious ^h^ | Not serious | Serious ^i^ | Both studies had co-interventions ^j^ | ART initiation increased under the intervention: in Kaplan (2016), 74% of 3411 patients initiated ART under the intervention compared to 67% of 3719 in the comparator group, RR 1.10 (1.07, 1.14); in Ogarkov (2016), 54% of 97 patients initiated ART under the intervention compared to 17% of 102 in the comparator group, RR 3.22 (1.92, 5.41). | ⊕🌕🌕🌕  Very low | Critical |

1. Rocha (2011) was conducted in the community in Peru from 2007-2010 and included all TB patients and their household contacts living in eight shantytowns. Patients were educated and received psychological counselling, principally for depression and substance abuse.
2. Quasi-experimental (historical control).
3. Serious risk of bias due to confounding and missing data.
4. Cannot asses for a single study.
5. Patient financial support.
6. Kaplan (2016) was conducted in TB clinics located within primary health care facilities in South Africa and included all newly registered TB patients at participating facilities from 2013-2014 (staggered). Ogarkov (2016) was conducted at a TB hospital in Russia and included all TB patients >15 years newly diagnosed with HIV.
7. Serious risk of bias due to confounding only, no adjustments (Ogarkov); moderate risk of bias due to missing data, some missingness for mortality outcome and patients excluded if they transferred between clinics (Kaplan).
8. Kaplan (2016) was conducted at clinics in South Africa, patients were educated on TB-HIV coinfection/care using flipcharts, had dedicated adherence counsellors and lay health care workers, and trained all health care workers. Ogarkov (2016) was conducted in a hospital in Russia, and patients received an educational message about ART alongside major operational improvements including prioritization of ART in TB-HIV coinfected patients through weekly cohort reviews, expedited CD4 and VL tests. Studies have non-overlapping CIs.
9. Sample size and number of events is low in one study (Ogarkov).
10. Dedicated personnel (Kaplan), health care worker training (Kaplan), major operational improvements (Ogarkov).

**INTERVENTION 3** : **Dedicated personnel**

| **Certainty assessment** | | | | | | | **Impact** | **Certainty** | **Importance** |
| --- | --- | --- | --- | --- | --- | --- | --- | --- | --- |
| **No of studies** | **Study Design** | **Risk of Bias** | **Inconsistency** | **Indirectness** | **Imprecision** | **Other considerations** |  |  |  |
| HIV TESTING | | | | | | | | | |
| 1^a^ | Observational ^b^ | Unable to assess ^c^ | Not serious ^d^ | Not serious | Not serious | Study had a co-intervention that is considered standard of care ^e^ | HIV testing increased under the intervention: 93.4% of 258 patients tested for HIV under the intervention compared to 34.1% of 296 in the comparator group, RR 2.73 (2.33, 3.22). | ⊕🌕🌕🌕  Very low | Critical |
| ART INITIATION | | | | | | | | | |
| 3^f^ | Observational and RCT ^g^ | Serious ^h^ | Serious ^i^ | Not serious | Serious ^j^ | Two studies had co-interventions, some considered standard of care ^k^ | ART initiation increased in one study under the intervention, and did not significantly increase in two other studies: in Chukwuka (2011), 19.6% of 92 patients initiated ART under the intervention compared to 8.9% of 56 in the comparator group, RR 2.19 (0.86, 5.57); in Kaplan (2016), 74% of 3411 patients initiated ART under the intervention compared to 67% of 3749 in the comparator, RR 1.10 (1.07, 1.14); in Kufa (2017), 39.3% of 224 patients initiated ART under the intervention compared to 38.8% of 160 in the comparator, RR 0.99 (0.64, 1.54). | ⊕🌕🌕🌕  Very low | Critical |

1. Chukwuka (2011) was conducted at a TB clinic within a hospital in Nigeria and included all registered TB patients. A dedicated HCT counsellor tested TB patients for HIV.
2. Quasi-experimental study (historical control).
3. Abstract. Not enough information to assess.
4. Cannot assess for a single study.
5. Systematic HIV testing of all TB patients.
6. Chukwuka (2011) was conducted at a TB clinic within a hospital in Nigeria and included all registered TB patients. Kaplan (2016) was conducted in TB clinics located within primary health care facilities in South Africa from 2013-2014 (staggered) and included all newly registered TB patients at participating facilities. Kufa (2017) was conducted at primary health care facilities in South Africa from 2011-2014 and included all patients >18 years newly diagnosed with TB, HIV or both.
7. Quasi-experimental (Chukwuka, Kaplan). Cluster RCT (Kufa).
8. Unable to assess (Chukwuka), moderate risk of bias due to missing data, some missingness for mortality outcome and patients excluded if they transferred between clinics (Kaplan), high due to blinding and confounding due to remaining baseline imbalances (Kufa).
9. Chukwuka (2011) was conducted in a hospital in Nigeria, and a dedicated HCT counsellor tested TB patients for HIV; systematic HIV testing was introduced as part of the intervention. Both Kaplan (2016) and Kufa (2017) were conducted at primary health care facilities in South Africa, but dedicated personnel were diverse (Kaplan - adherence counsellors and lay health workers, Kufa - professional nurses), and they performed diverse tasks (Kaplan - educated patients on TB-HIV coinfection/care using flipcharts, Kufa - served as TB-HIV integration and TB screening officers to support a variety of TB-HIV integration efforts).
10. CI crosses the null in two studies (Chukwuka, Kufa). Sample size and number of events are also low in both studies.
11. Patient education and counselling (Kaplan), health care worker training (Kaplan), systematic HIV testing (Chukwuka).

**INTERVENTION 4**: **Patient peer support**

| **Certainty assessment** | | | | | | | **Impact** | | | | **Certainty** | **Importance** |
| --- | --- | --- | --- | --- | --- | --- | --- | --- | --- | --- | --- | --- |
| **No of studies** | **Study Design** | **Risk of Bias** | **Inconsistency** | **Indirectness** | **Imprecision** | **Other considerations** |  |  |  |  |  |  |
| HIV TESTING | | | | | | | | | | | | |
| 0 |  |  |  |  |  |  |  |  |  |  |  |  |
| ART INITIATION | | | | | | | | | | | | |
| 1^a^ | Observational ^b^ | Serious ^c^ | Not serious ^d^ | Not serious | Serious ^e^ | Study had co-interventions that are considered standard of care ^f^ | ART initiation did not appear to increase under the intervention: 88.6% of 79 patients initiated ART under the intervention compared to 94.4% of 89 in the comparator group, RR 0.94 (0.85, 1.03). | | | | ⊕🌕🌕🌕  Very low |  |

1. Courtney-Quirk (2018) - intervention arm 2 was conducted at standalone TB clinics in Tanzania and included all TB patients newly diagnosed with HIV at participating clinics. Volunteer patient peers supported TB-HIV integration activities.
2. Modified stepped-wedge design (historical control).
3. Serious risk of bias due to confounding, no adjustment or identification of potential confounding variables.
4. Cannot assess inconsistency for one study.
5. CI crosses the null. Sample size is also low.
6. Health care worker training, operational improvements (introduction of an HIV register and ART logbook).

**INTERVENTION 5**: **Patient financial support**

| **Certainty assessment** | | | | | | | **Impact** | **Certainty** | **Importance** |
| --- | --- | --- | --- | --- | --- | --- | --- | --- | --- |
| **No of studies** | **Study Design** | **Risk of Bias** | **Inconsistency** | **Indirectness** | **Imprecision** | **Other considerations** |  |  |  |
| HIV TESTING | | | | | | | | | |
| 1^a^ | Observational ^b^ | Serious ^c^ | Not serious ^d^ | Not serious | Not serious | Study had a co-intervention ^e^ | HIV testing increased under the intervention: 96.9% of 318 TB patients tested for HIV under the intervention compared to 30.6% of 72 in the comparator group, RR 3.17 (2.24, 4.49). | ⊕🌕🌕🌕  Very low |  |
| ART INITIATION | | | | | | | | | |
| 0 |  |  |  |  |  |  |  |  |  |

1. Rocha (2011) was conducted in the community in Peru from 2007-2010 and included all TB patients and their household contacts living in eight shantytowns. Patients received vocational/training on income generation, micro-enterprise, and food/cash transfers.
2. Quasi experimental study (historical control).
3. Serious risk of confounding and serious risk of bias due to missing data.
4. Cannot assess inconsistency for a single study.
5. Patient education and counselling.

### PICO 2: TB case detection / ATT initiation in PLHIV

**INTERVENTION 1**: Co-located TB Screening and Testing (Same Facility)

| Certainty assessment | | | | | | | No of patients | | Effect | | Certainty | Importance |
| --- | --- | --- | --- | --- | --- | --- | --- | --- | --- | --- | --- | --- |
| **No of studies** | **Study Design** | **Risk of Bias** | **Inconsistency** | **Indirectness** | **Imprecision** | **Other considerations** | **Intervention** | **Control** | **Relative (95% CI)** | **Absolute (95% CI)** |  |  |
| TB CASE DETECTION | | | | | | | | | | | | |
| 1^a^ | Observational ^b^ | Not serious | Not serious ^c^ | Not serious | Serious ^d^ | Study had co-interventions that are considered standard of care ^e^ | 66/402 (16.4%) | 40/380 (10.5%) | 1.56 (1.08-2.25) | **59 more per 1,000** (from 8 more to 131 more) | ⊕  Very Low | Critical |
| ATT INITIATION | | | | | | | | | | | | |
| 1^a^ | Observational ^b^ | Not serious | Not serious ^c^ | Not serious | Not serious | Study had co-interventions that are considered standard of care ^e^ | 561/565 (99.3%) | 297/297 (100%) | 0.99 (0.99-1.00) | **1 fewer per 1,000** (from 0 fewer to 1 fewer) | ⊕⊕  Low | Critical |

1. Agarwal (2018) was conducted in standalone HIV clinics in Ukraine from 2012-2015 and included all patients >18 years attending facilities offering inpatient/intensive treatment;
2. Non-randomized cluster-controlled before-and-after study. TB screening and testing co-located with HIV care services.
3. Cannot assess for a single study.
4. Low number of events with low sample size (TB detected in only 66 of 402 participants).
5. Health care worker training, major operational improvements (creation of electronic data management system).

**INTERVENTION 2**: Patient Education and Counselling

| **Certainty assessment** | | | | | | | **Impact** | **Certainty** | **Importance** |
| --- | --- | --- | --- | --- | --- | --- | --- | --- | --- |
| **No of studies** | **Study Design** | **Risk of Bias** | **Inconsistency** | **Indirectness** | **Imprecision** | **Other considerations** |  |  |  |
| TB CASE DETECTION | | | | | | | | | |
| 3^a^ | Observational ^b^ | Serious^c^ | Serious ^d^ | Not serious | Serious ^e^ | TB and HIV services were co-located in the control and intervention arms of two studies. Studies had diverse co-interventions ^f^ | TB case detection increased under the intervention in one study and appeared to increase in two others: in Kanara (2008), TB was detected in 13.3% of 751 patients under the intervention compared to 8.7% of 1228 in the comparator group, RR 1.53 (1.18, 1.98); in Hermans S (2012), TB was detected in 1.7% of 10525 patients under the intervention compared to 1.4% of 9931 in the comparator group, RR 1.22 (0.98, 1.52); in Mathebula (2020), TB was detected in 12.1% of 993 patients in the intervention compared to 9.7% of 870 in the comparator group, RR 1.25 (0.96, 1.63). | ⊕  Very Low | Critical |
| ATT INITIATION | | | | | | | | | |
| 1^g^ | Observational ^b^ | Serious^h^ | Not serious ^i^ | Not serious | Serious ^j^ | TB and HIV services were co-located in the control and intervention arms..Study had a co-intervention ^i^ | ATT initiation did not appear to increase under the intervention: 91.7% of 180 TB-HIV coinfected patients receiving the intervention initiated ATT compared to 94.2% of 139 patients in the comparator group, RR 0.97 (0.92, 1.03). | ⊕  Very Low | Critical |

1. Hermans S (2012) was conducted at a standalone HIV clinic in Uganda in 2010 and included all adult patients attending the clinic who were not already diagnosed with TB and on TB treatment. Kanara (2008) was conducted in standalone TB and HIV clinics in Cambodia in 2005 and included all HIV and TB patients registered at participating facilities. Mathebula (2020) was conducted at a standalone HIV clinic in Botswana in 2013-2014 and included all new HIV clinic attendees aged over 12 years who screened positive for TB.
2. Quasi-experimental study/ies (historical control).
3. Serious risk of bias due to confounding (Hermans S, Kanara) and missing data (Hermans S). Kanara 2008, quasi-experimental study comparing PEC to historical control. Patients educated on the risk of TB for all PLHIV.
4. Studies were conducted in standalone clinics but in diverse countries/regions (Hermans S – Uganda, Mathebula – Botswana, Kanara – Cambodia) and implemented education on diverse topics and in diverse ways - Hermans S (2012) introduced peer supporters to announce information on TB, TB-HIV coinfection and TB screening questions to encourage patients in queue to self-identify for TB screening and testing, Kanara (2008) gave existing VCCT counsellors a written script to educate patients on the risk of TB for all PLHIV, whereas Mathebula (2020) used existing nurses to educate and assist patients on sputum induction technique. Studies also implemented diverse other interventions including operational improvements concurrent with patient education and counselling (Hermans S – peer support, Kanara - data management forms and staff education meetings, Mathebula - health care worker training and mentorship,, sputum collection job aids, logbooks and supervision/monitoring of screening/ documentation activities related to TB-HIV care.
5. CI crossed the null in two studies (Kanara, Mathebula). Both studies also had low sample sizes and low number of events.
6. Co-interventions included: patient peer support (Hermans S), health care worker training (Kanara, Mathebula), operational improvements (Kanara, Mathebula). In control and intervention arms, TB testing services were co-located with HIV services (Mathebula, same facility) and TB testing and TB treatment services were co-located with HIV services (Hermans S, same facility and provider).
7. Hermans S (2012) was conducted at a standalone HIV clinic in Uganda in 2010 and included all adult patients attending the clinic who were not already diagnosed with TB and on TB treatment. Patients waiting in queue were educated about TB, TB-HIV coinfection and TB screening questions to encourage them to self-identify for TB screening and testing.
8. Serious risk of bias due to confounding and missing data.
9. Cannot assess for a single study.
10. CI crosses the null. Low sample size.
11. Co-intervention included: patient peer support. In control and intervention arms, TB testing and TB treatment services were co-located with HIV services (same facility and provider).

**INTERVENTION 3**: Dedicated Personnel

| **Certainty assessment** | | | | | | | **Impact** | | | | | **Certainty** | **Importance** |
| --- | --- | --- | --- | --- | --- | --- | --- | --- | --- | --- | --- | --- | --- |
| **No of studies** | **Study Design** | **Risk of Bias** | **Inconsistency** | **Indirectness** | **Imprecision** | **Other considerations** |  |  |  |  |  |  |  |
| TB CASE DETECTION | | | | | | | | | | | | | |
| 2^a^ | RCT ^b^ | Serious ^c^ | Not serious ^d^ | Not serious | Not serious | TB and HIV services were co-located in control and intervention arms. Implemented co-interventions, some considered standard of care ^e^ | | TB case detection increased in both intervention arms of this study: TB was detected in 5% of 1724 and 6% of 4093 patients under the intervention, compared to 1.5% of 8621 patients in the comparator groups, RR 3.33 (2.55, 4.36) and RR 3.98 (3.23, 4.92), respectively for each intervention arm. | | | | ⊕⊕  Low | Critical |
| ATT INITIATION | | | | | | | | | | | | | |
| 0 |  |  |  |  |  |  |  | |  |  |  |  |  |

1. Auld (2020) contributed two intervention arms, both were conducted at an HIV clinic within a primary health care facility in Botswana and included all new HIV clinic attendees aged over 12 years who newly initiated ART or after study enrollment. Dedicated personnel (nurses) supported TB screening and testing services. Both intervention arms received the same intervention. In the first arm, patients were diagnosed with sputum smear microscopy. In the second arm, patients were diagnosed with GeneXpert.
2. Stepped-wedge cluster randomized trial.
3. Lack of blinding and confounding, no adjustment for cluster-level imbalance for TB case detection.
4. Cannot assess for a single study.
5. Co-interventions included: systematic TB screening of PLHIV at every clinic visit, health care worker training, operational improvements (checklists/job aids, staff supervisory visits). In control and intervention arms, TB testing was co-located with HIV services (same facility).

**INTERVENTION 4**: Peer Support

| **Certainty assessment** | | | | | | | **Impact** | **Certainty** | **Importance** |
| --- | --- | --- | --- | --- | --- | --- | --- | --- | --- |
| **No of studies** | **Study Design** | **Risk of Bias** | **Inconsistency** | **Indirectness** | **Imprecision** | **Other considerations** |  |  |  |
| TB CASE DETECTION | | | | | | | | | |
| 1^a^ | Observational ^b^ | Serious ^c^ | Not serious ^d^ | Not serious | Serious ^e^ | TB and HIV services were co-located in the control and intervention arms. Study had a co-intervention ^e^ | TB case detection did not significantly increase under the intervention: TB was detected in 1.7% of 10525 patients under the intervention compared to 1.4% of 9931 in the comparator group, RR 1.22 (0.98, 1.52). | ⊕  Very Low | Critical |
| ATT INITIATION | | | | | | | | | |
| 1^a^ | Observational ^b^ | Serious ^c^ | Not serious ^d^ | Not serious | Serious ^e^ | TB and HIV services were co-located in the control and intervention arms. Study had a co-intervention ^e^ | ATT initiation did not appear to increase under the intervention: 91.7% of 180 patients initiated ATT under the intervention compared to 94.2% of 139 patients in the comparator group, RR 0.97 (0.92, 1.03). | ⊕  Very Low | Critical |

1. Hermans S (2012) was conducted at a standalone HIV clinic in Uganda in 2010 and included all adult patients attending the clinic who were not already diagnosed with TB and on TB treatment. Patient peer supporters delivered patient education.
2. Quasi-experimental study (historical control).
3. Serious risk of bias due to confounding and missing data.
4. Unable to assess for a single study.
5. CI crosses the null. Low sample size.
6. Co-intervention included: patient education and counselling. In control and intervention arms, TB testing and TB treatment services were co-located with HIV services (same facility and provider).
