# Supplementary material for: Interventions to improve linkage along the HIV-tuberculosis care cascades in low- and middle-income countries: A systematic review and meta-analysis
Source: PLoS One. 2022 May 12;17(5):e0267511. doi: 10.1371/journal.pone.0267511 (PMC9098064; doi:10.1371/journal.pone.0267511)
Supplement: S6 File — (DOCX) [file pone.0267511.s006.docx]

## Supplemental 5: Secondary Outcomes

### Table 1. Time to ART initiation

| **Ref** | **Study** | **Intervention** | **Population**  **(Control)** | **Median days**  **(Control)** | **IQR**  **(Control)** | **Population (int.)** | **Median days (int.)** | **IQR**  **(int.)** | **Conclusion** |
| --- | --- | --- | --- | --- | --- | --- | --- | --- | --- |
| 6 | Herce, 2018 | HCW training and mentorship; systematic and provider-level co-located HIV testing (for PWTB); provider-level co-located ART initiation; major operational improvements including dedicated ART clinic days and synchronized TB and HIV patient follow-up by dedicated TB-HIV personnel; peer-led patient education talks  **Co-location (F, P – ST and Tx)**  + Educ/Couns, Dedic Person, Peer Supp + HCW Train, Oper Improv, Syst HIV T | 103 | 264 | Not reported | 117 | 78 | Not reported | Intervention decreased time to ART |
| 8 | Hermans SM, 2012 | Provider-level co-located HIV testing (for PWTB) delivered by trained, dedicated personnel (peer supporters/lay HCWs); facility-level co-located treatment; major operational improvements including discussion of “difficult cases” at weekly team meetings, placement of ART initiation guides in clinic files, and phone-tracing to prevent loss to follow-up  **Co-location (F – ST and Tx; P – Tx only)**  + Dedic Person, Peer Supp + HCW Train, Oper Improv | 243 | 103 | Not reported | 229 | 45 | Not reported | Intervention decreased time to ART |
| 13 | Kerschberger, 2012 | Systematic and provider-level co-located HIV testing (for PWTB); minor operational improvements including combined health information system, patient filing system (with medical notes, screening tools, prescription charts) and monitoring/evaluation; provider-level co-located ART initiation; oversight of integrated program by dedicated personnel (facility manager)  **Co-location (F, P – ST and Tx)**  + HCW Train, Oper Improv, Syst HIV T | 100 | 147 | (95% CI)  85-188 | 88 | 75 | (95% CI)  52-119 | Intervention decreased time to ART |
| 15 | Louwagie, 2012 | Facility- level co-location of ART initiation.  **Co-location (F – Tx only)** | 104 | 67 | 35-109.5 | 74 | 67.5 | 53.92 | Intervention did not impact time to ART |
| 19 | Ogarkov, 2016 | Major operational improvements including expedition of CD4 cell count and viral load testing + administrative prioritization of ART requests for co-infected patients through weekly cohort reviews of all PLHIV; patient education tailored to people with HIV and TB  **Educ/Couns**  + Oper Improv | 14 | 175 | 104-281 | 44 | 67 | 47-104 | Intervention decreased time to ART |
| 20 | Owiti, 2015 | Facility-level co-located testing and treatment  **Co-location (F – ST and Tx)** | 178 | 42 | 27-82 | 20 | 40 | 15-75 | Intervention did not impact time to ART |
|  | Owiti, 2015 | Facility-level co-located testing, provider-level co-located treatment  **Co-location (F, P – ST, P – Tx only)** | 178 | 42 | 27-82 | 77 | 29 | 16-69 | Intervention decreased time to ART |
|  | Owiti, 2015 | Provider-level co-located testing and treatment  **Co-location (F, P – ST and Tx)** | 178 | 42 | 27-82 | 99 | 35 | 20-64 | Intervention decreased time to ART |

ART = antiretroviral treatment; F = co-location at the level of the same facility; HCW Train = healthcare worker training in TB-HIV; Oper Impro = operational improvements to support TB-HIV care; P = co-location at the level of the same provider; ST = co-located screening and/or testing of HIV and/or TB; Syst HIV T = systematic HIV testing under an opt-out provider initiated approach; Syst TB ST = systematic TB screening and testing using a standardized tool; Tx = co-located treatment of ART and ATT.

### Table 2. Mortality

| **Ref** | **Study** | **Intervention** | **Population** | **Population (Control)** | **Mortality percentage of control (95% CI)** | **Population (int.)** | **Mortality percentage of int. (95% CI)** | **RR**  **(95% CI)** | **Conclusion** |
| --- | --- | --- | --- | --- | --- | --- | --- | --- | --- |
| 1 | Agarwal, 2018 (HIV Centers) | Facility-level co-located and systematic HIV testing (for PWTB) and systematic TB screening for (PLHIV); HCW training in caring for co-infected patients; major operational improvements including development of electronic data-management system and other capacity building initiatives to institutionalize best practices in TB-HIV care  **Co-location (F – ST only)**  + HCW Train, Oper Improv, Syst HIV T, Syst TB ST | Co-infected | --- | **---** | **---** | **---** | 0.78 (0.46-1.36) ^a^ | Intervention did not impact mortality |
|  | Agarwal, 2018 (TB Centers) |  | TB cohort (not all HIV+) | --- | **---** | **---** | **---** | 1.32 (0.83-2.12) ^a^ | Intervention did not impact mortality |
| 2 | Ansa, 2014 (int 1) | **Int 1**: Facility-level co-located HIV testing  **Co-location (F – ST only)** | TB cohort (not all HIV+) | 247 | 14.9 (11.1-19.9) | 121 | 21.5 (15.1-29.6) | 1.43 (0.91-2.25) | Intervention did not impact mortality |
|  | Ansa, 2014 (int 2) | **Int 2**: provider-level co-located HIV testing, and ART initiation  **Co-location (F, P – ST and Tx)** | TB cohort (not all HIV+) | 247 | 14.9 (11.1-19.9) | 199 | 18.1 (13.4-24.0) | 1.21 (0.79-1.84) | Intervention did not impact mortality |
| 3 | Auld, 2020 (int 2) | **Int 1:**  Systematic TB screening for PLHIV at all visits (“intensified case finding”); HCW training (clinic and lab personnel); support from dedicated personnel (additional nurses); minor operational improvements including checklists/ job aids to standardize implementation, and regular supervisory visits.  **Int 2**: Int 1 + sputum smear microscopy replaced with Gene Xpert  **Dedic Person**  + HCW Train, Oper Improv, Syst TB ST | HIV cohort (not all TB+) | 8980 | 5.1 (4.7-5.6) | 4215 | 3.1 (2.3-3.9) | 0.76 (0.61-0.95) ^b^ | **Intervention decreased mortality** |
| 8 | Hermans SM, 2012 | Provider-level co-located HIV testing (for PWTB) delivered by trained, dedicated personnel (peer supporters/lay HCWs); facility-level co-located treatment; major operational improvements including discussion of “difficult cases” at weekly team meetings, placement of ART initiation guides in clinic files, and phone-tracing to prevent loss to follow-up  **Co-location (F – ST and Tx; P – Tx only)**  + Dedic Person, Peer Supp + HCW Train, Oper Improv | Co-infected | 346 | 2.9 (1.6-5.2) | 366 | 15.2 (11.9-19.4) | 5.29 (2.75-10.21) | Intervention did not impact mortality |
| 9 | Huerga, 2010 | Facility-level co-located (non-systematic) HIV testing and ART initiation at the TB clinic, delivered by three additional dedicated personnel (clinical officer, nurse and counsellor); patient education on HIV prevention  **Co-location (F – ST and Tx)**  + Educ/Couns, Dedic Person | TB cohort (not all HIV+) | 409 | 7.6 (5.4-10.6) | 437 | 10.3 (7.8-13.5) | 1.36 (0.88-2.10) | Intervention did not impact mortality |
| 10 | Ikeda, 2014 | Extensive HCW training in HIV/TB co-infection (40% of providers received additional training in HIV integrated care through national 8-month diploma program); systematic and provider-level co-located HIV testing; facility-level co-located ART initiation  **Co-location (F – ST and Tx; P – ST only)**  + HCW Train, Syst HIV T | Co-infected | 98 | **77.6 (68.3-84.7)** | **155** | **27.1 (20.7-34.6)** | **0.35 (0.26-0.46)** | **Intervention decreased mortality** |
| 12 | Kaplan, 2016 | In-clinic TB educational sessions for all TB patients and HIV educational sessions for HIV-positive TB patients (patient education) performed by dedicated staff (adherence counsellors/ lay HCWs), following HCW training  **Educ/Couns** + **Dedic Person**  + HCW Train | Co-infected | 5691 | 6.1 (5.5-6.7) | 5140 | 5.3 (4.7-5.9) | 0.98 (0.80-1.21) ^c^ | Intervention did not impact mortality |
| 13 | Kerschberger, 2012 | Systematic and provider-level co-located HIV testing (for PWTB); minor operational improvements including combined health information system, patient filing system (with medical notes, screening tools, prescription charts) and monitoring/evaluation; provider-level co-located ART initiation; oversight of integrated program by dedicated personnel (facility manager)  **Co-location (F, P – ST and Tx)**  + HCW Train, Oper Improv, Syst HIV T | Co-infected | 100 | 9.0 (4.8-16.2) | 88 | 7.9 (3.9-15.5) | 0.88 (0.34-2.27) | Intervention did not impact mortality |
| 23 | Van Rie, 2014 | Task-shifting of CD4-stratified ART initiation from clinicians to TB nurses; provider-level co-location of ART initiation.  **Co-location (P – Tx only)**  + Task Shift | Co-infected | 373 | **20.1 (16.4-24.5)** | 513 | **9.8 (7.5-12.6)** | **0.48 (0.35-0.68)** | **Intervention decreased mortality** |

ART = antiretroviral treatment F = co-location at the level of the same facility; HCW Train = healthcare worker training in TB-HIV; Oper Impro = operational improvements to support TB-HIV care; P = co-location at the level of the same provider; ST = co-located screening and/or testing of HIV and/or TB; Syst HIV T = systematic HIV testing under an opt-out provider initiated approach; Syst TB ST = systematic TB screening and testing using a standardized tool; Task shift = task-shifting of TB-HIV services from specialized to less specialized workers; Tx = co-located treatment of ART and ATT.

1. HR adjusted for difference-in-differences model, as calculated by authors
2. HR adjusted for age, sex, pregnancy status, weight, CD4 count, hemoglobin, and ART regimen, as calculated by authors.
3. HR adjusted for age, sex, HIV status, CD4 cell count, calendar year and type of TB (pulmonary or extra-pulmonary), as calculated by authors
