# Supplementary material for: Interventions to improve linkage along the HIV-tuberculosis care cascades in low- and middle-income countries: A systematic review and meta-analysis
Source: PLoS One. 2022 May 12;17(5):e0267511. doi: 10.1371/journal.pone.0267511 (PMC9098064; doi:10.1371/journal.pone.0267511)
Supplement: S7 File — (DOCX) [file pone.0267511.s007.docx]

## Supplemental 6: Interventions identified in control and intervention arms

| **Studies** | | | | **Interventions ^a^** | | | | | | | | | | | | |
| --- | --- | --- | --- | --- | --- | --- | --- | --- | --- | --- | --- | --- | --- | --- | --- | --- |
| **Ref** | **Author, year (arm)** | **Outcomes** | **Country** | **Co-location** | | | | **Educ/ Couns** | **Dedic Person** | **Peer Supp** | **Financ Supp** | **Standard of care strategies ^b^** | | | | |
|  |  |  |  | **Service** | | **Level** | |  |  |  |  |  |  |  |  |  |
|  |  |  |  | **ST** | **Tx** | **F** | **P** |  |  |  |  | **Task Shift** | **HCW Train** | **Oper Impro** | **Syst HIV T** | **Syst TB ST** |
| 1 | Agarwal, 2018 (Control) | HIVi, ARTi, TBcd, ATTi | Ukraine |  |  |  |  |  |  |  |  |  |  |  |  |  |
|  | Agarwal, 2018 (intervention) |  |  |  |  |  |  |  |  |  |  |  |  | ++ |  |  |
| 2 | Ansa, 2014 (Control) |  | Ghana |  |  |  |  |  |  |  |  |  |  |  |  |  |
|  | Ansa, 2014 (int 1) | HIVt, ARTi |  |  |  |  |  |  |  |  |  |  |  |  |  |  |
|  | Ansa, 2014 (int 2) |  |  |  |  |  |  |  |  |  |  |  |  |  |  |  |
| 3 | Auld, 2020 (Control) | TBcd | Botswana |  |  |  |  |  |  |  |  |  |  |  |  |  |
|  | Auld, 2020 (int 1) |  |  |  |  |  |  |  |  |  |  |  |  |  |  |  |
|  | Auld, 2020 (int 2) |  |  |  |  |  |  |  |  |  |  |  |  |  |  |  |
| 4 | Chukwuka, 2011 (Control) | HIVt, ARTi | Nigeria |  |  |  |  |  |  |  |  |  |  |  |  |  |
|  | Chukwuka, 2011 (intervention) |  |  |  |  |  |  |  |  |  |  |  |  |  |  |  |
| 5 | Courtenay-Quirk, 2018  (Control) | ARTi | Tanzania |  |  |  |  |  |  |  |  |  |  |  |  |  |
|  | Courtenay-Quirk, 2018  (int 2) |  |  |  |  |  |  |  |  |  |  |  |  |  |  |  |
| 6 | Herce, 2018 (Control) | ARTi | Zambia |  |  |  |  |  |  |  |  |  |  |  |  |  |
|  | Herce, 2018 (int – clinic A) |  |  |  |  |  |  |  |  |  |  |  |  | ++ |  |  |
|  | Herce, 2018 (int – clinic B) |  |  |  |  |  |  |  |  |  |  |  |  | ++ |  |  |
| 7 | Hermans S, 2012 (Control) | TBcd, ATTi | Uganda |  |  |  |  |  |  |  |  |  |  |  |  |  |
|  | Hermans S, 2012 (intervention) |  |  |  |  |  |  |  |  |  |  |  |  |  |  |  |
|  | | | | | | | | | | | | | | | | |
| 8 | Hermans SM, 2012 (Control) | ARTi | Uganda |  |  |  |  |  |  |  |  |  |  |  |  |  |
|  | Hermans SM, 2012 (intervention) |  |  |  |  |  | Tx |  |  |  |  |  |  | ++ |  |  |
| 9 | Huerga (Control) | ARTi | Kenya |  |  |  |  |  |  |  |  |  |  |  |  |  |
|  | Huerga (intervention) |  |  |  |  |  |  |  |  |  |  |  |  |  |  |  |
| 10 | Ikeda, 2014 (Control) | ARTi | Guatemala |  |  |  |  |  |  |  |  |  |  |  |  |  |
|  | Ikeda, 2014 (intervention) |  |  |  |  |  | ST |  |  |  |  |  |  |  |  |  |
| 11 | Kanara, 2008 (Control) | HIVt, TBcd | Cambodia |  |  |  |  |  |  |  |  |  |  |  |  |  |
|  | Kanara, 2008 (intervention) |  |  |  |  |  |  | TBcd |  |  |  |  |  |  |  |  |
| 12 | Kaplan, 2016 (Control) | ARTi | South Africa |  |  |  |  |  |  |  |  |  |  |  |  |  |
|  | Kaplan, 2016 (intervention) |  |  |  |  |  |  |  |  |  |  |  |  |  |  |  |
| 13 | Kerschberger, 2012 (Control) | ARTi | South Africa |  |  |  |  |  |  |  |  |  |  |  |  |  |
|  | Kerschberger, 2012 (intervention) |  |  |  |  |  |  |  |  |  |  |  |  |  |  |  |
| 14 | Kufa, 2018 (Control) | ARTi | South Africa |  |  |  |  |  |  |  |  |  |  |  |  |  |
|  | Kufa, 2018 (intervention) |  |  |  |  |  |  |  |  |  |  |  |  |  |  |  |
| 15 | Louwagie, 2012 (Control) | ARTi | South Africa |  |  |  |  |  |  |  |  |  |  |  |  |  |
|  | Louwagie, 2012 (intervention) |  |  |  |  |  |  |  |  |  |  |  |  |  |  |  |
| 16 | Mathebula, 2020 (Control) | TBcd | Botswana |  |  |  |  |  |  |  |  |  |  |  |  |  |
|  | Mathebula, 2020  (intervention) |  |  |  |  |  |  |  |  |  |  |  |  |  |  |  |
|  | | | | | | | | | | | | | | | | |
| 17 | Mwinga, 2008 (Control) | HIVt, ARTi | Zambia |  |  |  |  |  |  |  |  |  |  |  |  |  |
|  | Mwinga, 2008 (int 1) |  |  |  |  |  |  |  |  |  |  |  |  |  |  |  |
|  | Mwinga, 2008 (int 2) |  |  |  |  |  |  |  |  |  |  |  |  |  |  |  |
| 18 | Nateniyom, 2008 (Control) | HIVt | Thailand |  |  |  |  |  |  |  |  |  |  |  |  |  |
|  | Nateniyom, 2008 (intervention) |  |  |  |  |  |  |  |  |  |  |  |  |  |  |  |
| 19 | Ogarkov, 2016 (Control) | ARTi | Russia |  |  |  |  |  |  |  |  |  |  |  |  |  |
|  | Ogarkov, 2016 (intervention) |  |  |  |  |  |  |  |  |  |  |  |  | ++ |  |  |
| 20 | Owiti, 2015 (Control) | ARTi | Kenya |  |  |  |  |  |  |  |  |  |  |  |  |  |
|  | Owiti, 2015 (int 1) |  |  |  |  |  |  |  |  |  |  |  |  | ++ |  |  |
|  | Owiti, 2015 (int 2) |  |  |  |  |  | Tx |  |  |  |  |  |  | ++ |  |  |
|  | Owiti, 2015 (int 3) |  |  |  |  |  |  |  |  |  |  |  |  | ++ |  |  |
| 21 | Rocha, 2011 (Control) | HIVt | Peru |  |  |  |  |  |  |  |  |  |  |  |  |  |
|  | Rocha, 2011 (intervention) |  |  |  |  |  |  |  |  |  |  |  |  |  |  |  |
| 22 | Van Rie, 2008 (Control) |  | Democratic Republic of Congo |  |  |  |  |  |  |  |  |  |  |  |  |  |
|  | Van Rie, 2008 (int 1) | HIVt |  |  |  |  |  |  |  |  |  |  |  |  |  |  |
|  | Van Rie, 2008 (int 2) |  |  |  |  |  |  |  |  |  |  |  |  |  |  |  |
| 23 | Van Rie, 2014 (Control) | ARTi | Democratic Republic of Congo |  |  |  |  |  |  |  |  |  |  |  |  |  |
|  | Van Rie, 2014 (intervention) |  |  |  |  |  |  |  |  |  |  |  |  |  |  |  |

ARTi = ART initiation; ATTi = anti-TB treatment initiation; F = co-location at the level of the same facility; HCW Train = healthcare worker training in TB-HIV; HIVt = HIV test completion; Oper Impro = operational improvements to support TB-HIV care; P = co-location at the level of the same provider; ST = co-located screening and/or testing of HIV and/or TB; Syst HIV T = systematic HIV testing under an opt-out provider initiated approach; Syst TB ST = systematic TB screening and testing using a standardized tool; Task shift = task-shifting of TB-HIV services from specialized to less specialized workers; TBcd = TB case detection; Tx = co-located treatment of ART and ATT.

1. See Table 3 of main report for intervention details. It is noted when an intervention aimed to address only one of many outcomes addressed by the study (e.g., Kanara 2008). It also noted when an intervention or strategy was explicitly stated by authors as being included in the control and intervention arm/s (e.g., Auld 2020).
2. Strategies that are now recommended as standard of care are noted when implemented as part of an intervention package.
3. Major operational improvements are indicated by ++ (see Table 3 of main report for intervention details).
