# Supplementary material for: Interventions to improve linkage along the HIV-tuberculosis care cascades in low- and middle-income countries: A systematic review and meta-analysis
Source: PLoS One. 2022 May 12;17(5):e0267511. doi: 10.1371/journal.pone.0267511 (PMC9098064; doi:10.1371/journal.pone.0267511)
Supplement: S8 File — (DOCX) [file pone.0267511.s008.docx]

## Supplemental 7: Implementation considerations

### Table 1. Summary of implementation facilitators and barriers by outcome

| **Outcome** | **Facilitators** | **Barriers** |
| --- | --- | --- |
| HIVt | **Patients**   - Patient education about risk and management HIV/AIDS among PWTB, from peer-educators or providers [11]   **Providers**   - HCW training: standardized curriculum or tools (such as standardized script) on HIV counselling and testing (and how to counsel patients who initially refuse), linking with HIV services, risk of HIV among TB patients, use of rapid tests; also with a focus also on team-building between TB-HIV staff if not fully integrated [11,17] - Task-shifting of HIV testing from laboratory/high-level HCWs to lay or non-health professionals (community health workers – CHWs) - reduces wait time and increases acceptability to testing [17] - Multidisciplinary teams composed of laboratory, in-patient and out-patient staff involved in TB and HIV programmes to oversee implementation, with frequent team meetings between separate clinic staff [18] - Motivated, enthusiastic, invested TB programme staff who recognize the problem of HIV-associated TB and challenges around inefficient referral models [18] - Local feedback, trained data management staff at facility levels [1,2]   **Facilities/Programs**   - Availability of HIV testing within TB facility [18] - Opt-out testing [6] - Use of rapid tests for same-day results [1,17] - Participation of critical policy makers such as regional and provincial TB programme managers, hospital directors and MOPH officials [18] - Strong coordination between TB and HIV programmes and between clinical and public health staff and by intensified supervision, monitoring and evaluation [18] | **Patients**   - Patients believing they are not at risk of HIV – particularly a challenge among young, educated patients [18] - Long process of counselling and testing (in traditional “VCT” models) [2] - Concerns around confidentiality, convenient timing/location of testing [2, 20]   **Facilities/Programs**   - Tests requiring blood to be drawn [18] - Tests requiring written consent [18] - Supply-chain management issues: Unavailability of HIV tests, ARTs [1,22] |
| ARTi | **Patients**   - Treatment literacy which increase awareness among patients of the need for ART initiation during anti-TB treatment [12, 19] - Formation of active civil society of TB-affected households, with advocacy activities giving the group a collective voice in regional decisions influencing TB care [21] - Economic and nutritional incentives, social support programs [1,21]   **Providers**   - Review and monitoring of co-infected patient folders, sharing of lessons-learned and clinic-level best practices at frequent multidisciplinary team meetings [1, 6, 8, 12, 19] - Task-shifting – nurse-centered instead of physician-centered ART initiation and management [2] - Sensitizing staff pre-implementation to achieve adequate integration; a team convinced of the need for integration [6, 8, 13] - Changing management processes to clarify defined areas of accountability for health staff [13] - High quality, professional, compassionate staff [1] - Extra staff to absorb increases in co-infected patients and support TB staff overseeing TB clinic operations i.e. “extra nurse and a roving part time doctor” [6, 13]   **Facilities/Programs**   - One patient, one file, one appointment, one provider, one-stop shop services [6, 8, 13, 15, 17, 19, 20] - Enhanced individual-level electronic medical records to ensure sharing of data on HIV care and TB care with providers of TB and HIV services [1, 5, 17] - Adherence and social support interventions within integrated programs that mutually reinforce each other rather than competing for scarce resources [13, 20] - Increased efforts for infection control (if services are co-located); facilitated/mentored by infection control officer [13] - Improved CD4 cell count and viral load testing availability and speed of delivery [19] - Prioritized referrals for ART to speed up committee approval for TB patients [19] - Involvement of policy makers, facility in-charges, health workers, and other leadership in program planning from the outset; obtaining local buy-in and visible MOH support [6, 13, 20] - Use of local resources, favouring national ownership and sustainability [20] - Longitudinal technical support for organizational and logistical problem solving around patient flow and supply chain management for TB and HIV commodities [6, 8] - Adequate clinical space; room for new patient enrolments and daily medication dispensing, patient examination on ART/TB clinic days, and health commodity storage and private counselling space [6, 8] | **Patients**   - Poverty, despair, stigmatisation and isolation [21] - Patient concern of high pill burden, toxicities and perceived risk of death associated with multiple drug regimens [6] - Stigma of HIV-associated TB [1, 6] - Challenges in navigating system, wait-times, transportation costs, inability for patients to take time off work - especially for referral-based services [1, 6, 20]   **Providers**   - Overburdened staff leading to incomplete/unreturned referral forms; this may also prevent staff from adopting intended coordinator/mentorship roles, instead providing care directly to patients [6, 13, 14, 17, 20] - Human resource challenges of frequent staff shortages, scheduling issues, turnover, low motivation, reluctance to adopt intervention [6, 20] - Negative provider attitudes towards patients [1] - Staff misconceptions; i.e. clinicians’ fear that patients with severe TB infections will develop serious ARV side effects, such as immune reconstitution inflammatory syndrome (IRIS) and drug toxic events [6, 13, 20]   **Facilities/Programs**   - Supply-chain management issues – restricted unavailability of ARTs and other essential commodities [1, 2, 6, 8, 13] - Delay in return of HIV tests (i.e. non-rapid tests) [18] - Stigma associated with injection drug use; policies preventing authorisation of ARTs for IDUs [19] |
| TBcd | **Patients**   - Patient education/ “mental preparedness of clients” [1] - An understanding among patients that all PLHIV are at risk of TB and should be screened, to overcome misconceptions about who is at low/high-risk [11]   **Providers**   - Knowledgeable, professional, motivated doctors and other staff [1] - An understanding among PROVIDER that all PLHIV are at risk of TB and should be screened, to overcome misconceptions about who is at low/high-risk [11] - Resources for additional lay health care workers [3, 7]   **Facilities/Programs**   - Enhanced data collection forms /registers – integrating TB-HIV healthcare data, attached to single patient file [11] - TB screening questionnaires designed for ease of use that prompt providers to utilise them (e.g. use of screening checklist, reminders) and favour sensitivity over specificity [3, 11] - Timely diagnostic testing (i.e. spot sputum/sputum induction and real time microscopy [1] - Testing of all patients regardless of symptoms (although not always financially feasible) [3, 7] - Additional screening to “sensitize patient to report TB symptoms during formal encounter with HIV provider”, as clinicians are less likely to think of TB in stable patients [7] - Resources to absorb additional TB test requests (i.e. laboratories able to handle additional workload) [7] | **Patients**   - Patient demographics and misconceptions; i.e. patients “feeling well”, semi-skilled/skilled workers, younger are less likely to be tested for TB [11] - Cost-saving methods of screening, like “announcement” method, may be inefficient due to stigma in reporting symptoms, especially in front of peers [7] - Inability for PLHIV to produce adequate sputum sample [16]   **Providers**   - Lower skill level/enthusiasm of peer supporters leading to incomprehensible or unclear messaging; language barriers [3, 7, 11] - Short-staffed facilities [1]   **Facilities/Programs**   - TB testing expenses {Agarwal, 2018 #54} - Faulty/ unavailable TB testing equipment or substandard laboratory performance [1, 7] - Leaks in the ICF cascade before a TB diagnostic test is implemented; especially failure to implement the WHO-recommended symptom screen [3] - Difficulty in diagnosing TB, especially in PLHIV [1] - Long wait time to be seen in integrated clinics [7] - Lack of sputum induction equipment [16] |
| ATTi | **None reported** | **Patients**   - Perceived side effects of TB medicine side effects [1] - Inconvenience of daily travel to dispensary for treatment, long lines [1]   **Programs**   - Lack of space and capacity to meet client needs – unwelcoming clinic space (i.e. unsanitary conditions) [1] - Inability for clinic to “absorb” increases in TB diagnoses; supply chain management challenges with increased demand for medication, drug shortages [1, 7] |

ART = antiretroviral treatment; ARTi = ART initiation; ATTi = anti TB treatment initiation; HCW = health care worker; HIVt = HIV testing; TB= tuberculosis; TBcd = TB case detection

### Table 2. Summary of resources and cost requirements by study

| **Ref** | **Author, year** | **Intervention** | **Feasibility & financial/resource requirements** |
| --- | --- | --- | --- |
| 8 | Hermans SM, 2012 | **Co-location (F – ST and Tx; P – Tx only)**  + Dedic Person, Peer Supp + HCW Train, Oper Improv | The integrated clinic was set up with a minimal use of resources, of which the majority was used for the construction of the outdoor clinic specifically for improvement of infection control, an outcome which we could not measure. The additional cost to provide the integrated care service was minimal and consisted mainly of efforts to create training, clinic, monitoring & evaluation materials, and to train the HIV/TB clinic staff. |
| 10 | Ikeda, 2014 | **Co-location (F – ST and Tx; P – ST only)**  + HCW Train, Syst HIV T | Required infrastructure: Improved cross-air ventilation in waiting room, nurses' pre-exam room, psychology and adherence rooms. Exam rooms have air extraction systems, UVP filtration, and ultraviolet lighting. |
| 12 | Kaplan, 2016 | **Educ/Couns** + **Dedic Person**  + HCW Train | The new programme required the support of community care workers and adherence counsellors. For the most part, this was accomplished through upskilling community DOT workers and retraining facility-based counsellors. While the introduction of the model required extra resources, the integration of TB and HIV adherence support, the decongestion of the clinic and task shifting would have resulted in a decrease in the use of resources at a clinic level. |
| 13 | Kerschberger, 2012 | **Co-location (F, P – ST and Tx)**  + HCW Train, Oper Improv, Syst HIV T | Environmental controls focus on maximizing low maintenance natural ventilation and ensure appropriate directional flow in consultation rooms. Small infrastructural changes were implemented to ensure a minimum of 12 air changes per hour in every room; these include the installation of additional windows, wind-driven roof turbines, an outside sputum booth, and dry-wall partitioning to modify patient-flow. Personal respiratory protection consists of distribution of surgical masks to all coughing patients and N95 respirators to health care workers. Increased focus on infection control throughout the health sub-district of Khayelitsha was made possible thanks to the addition of an infection control officer. As the number of patients on ART increased, an extra nurse and a roving part time doctor were added (after the period considered in this analysis). |
| 17 | Mwinga, 2008 | **Co-location (F, P – ST only)**  + HCW Train, Syst HIV T | Nurse-counselors were given transportation allowances that enabled them to travel from their homes to the clinics; The room adjacent to the chest clinic was renovated for counseling; part-time nurse-counselors were hired to work at on-site volunteer counselling and testing (VCT) and were given transportation allowances that enabled them to travel from their homes to the clinics. |
| 18 | Nateniyom, 2008 | **Co-location (F – ST and Tx)**  + HCW Train, Oper Improv, Syst HIV T | Because most project activities were integrated into routine services, we believe that additional funding for training is the only major financial input required to implement this activity nationally. The project was planned centrally but included participation of critical policy makers such as regional and provincial TB programme managers, hospital directors and MOPH officials. |
| 19 | Ogarkov, 2016 | **Educ/Couns**  + Oper Improv | The bundle of health system initiatives had no additional cost for the patients. The bulk of costs associated with the intervention were for labour re-organization and task shifting, which favour sustainability, but formal cost analyses were not performed. |
| 20 | Owiti, 2015 | **Co-location (F, P – ST and Tx)**  + Educ/Couns, Dedic Person + HCW Train | Implementation of intervention required (re)alignment of resources: - Rooms (for clinical care and other services) - Filing systems and record keeping - Pharmacy services (CPT, ART, TB and other drug refills) - Communication platforms - TB-HIV clinical care and consultations - HIV testing - Synchronized follow-up appointments - Personnel for nutrition services, social support services, adherence and disclosure counselling, outreach and patient tracing  - Key infection control practices |
| 21 | Rocha, 2011 | **Educ/Couns** + **Financ Supp** | Income increased for 3.2% of participating households as a result of vocational training, which cost an average of US$86 per person trained. Food and cash transfers constitute an important part of most project activities and have averaged US$160 in value per household (42% of per capita, 10% of TB-affected household median income). Food (23% of transfer value) is provided at all project events, and food packages are provided in patients' homes. Cash transfers have averaged 13% of transfer value for TB diagnosis and treatment costs supplementary to the main aspects of TB care, which are provided free by the NTP; 25% of transfer value for TB-related transport expenses; and 39% of transfer value for poverty reduction (excluding microcredit loans). |

ART = antiretroviral treatment; CPT = cotrimoxazole preventive therapy; Dedic Person = dedicated personnel; Educ/Couns = patient education/counselling; Financ Supp = patient financial support; HCW train = healthcare worker training; Oper Improv = operational improvements; PHF = primary healthcare facility; Peer Supp = patient peer support; Syst HIV T = systematic HIV testing; Syst TB ST = systematic TB screening and testing; Task Shift = task-shifting’ TB = tuberculosis.

### Table 3. Cost Analysis

| **Ref** | **Study, year** | **Intervention** | **Description of cost analysis** |
| --- | --- | --- | --- |
| 7 | Hermans S, 2012 | **Educ/Couns** + **Peer Supp** | By adding up all costs involved with the screening and the diagnosis of TB suspects before and after ICF implementation and then dividing this amount by the numbers of TB cases, we found that the cost per TB case found almost doubled from 12.29 USD pre-ICF to 21.80 USD post-ICF (Table 2). The greatest cost difference was incurred by the increase in numbers of investigations (sputum smears and chest X-rays) and the additional cost of the screening staff. Costs increased by 130% and TB cases identified by 30%. |

Educ/Couns = patient education/counselling; ICF = intensive case finding; Peer Supp = patient peer support.

## References (included studies)

1. **Agarwal** S, Brodish P, Brugh K, Charyeva Z, Curtis S, Fehringer J, et al. MEASURE Evaluation. Strengthening tuberculosis control in Ukraine: Evaluation of the impact of TB-HIV integration strategy on treatment outcomes. Chapel Hill, NC: University of North Carolina, 2018.

2. **Ansa** GA, Walley JD, Siddiqi K, Wei X. Delivering TB/HIV services in Ghana: a comparative study of service delivery models. Transactions of the Royal Society of Tropical Medicine & Hygiene. 2014;108(9):560-7.

3. **Auld** AF, Agizew T, Mathoma A, Boyd R, Date A, Pals SL, et al. Effect of tuberculosis screening and retention interventions on early antiretroviral therapy mortality in Botswana: A stepped-wedge cluster randomized trial. BMC Medicine. 2020;18(19).

4. **Chukwuka** CN, E; Onyedum, C. TB HIV Collaboration: What is the actual cost. Chest. 2011;140 (MeetingAbstracts).

5. **Courtenay-Quirk** C, Pals S, Howard AA, Ujamaa D, Henjewele C, Munuo G, et al. Increasing partner HIV testing and linkage to care in TB settings: findings from an implementation study in Pwani, Tanzania. AIDS Care - Psychological and Socio-Medical Aspects of AIDS/HIV. 2018;30(12):1600-4.

6. **Herce** ME, Morse J, Luhanga D, Harris J, Smith HJ, Besa S, et al. Integrating HIV care and treatment into tuberculosis clinics in Lusaka, Zambia: Results from a before-after quasi-experimental study. BMC Infectious Diseases. 2018;18(536).

7. **Hermans S**, Nasuuna E, van Leth F, Byhoff E, Schwarz M, Hoepelman A, et al. Implementation and effect of intensified case finding on diagnosis of tuberculosis in a large urban HIV clinic in Uganda: a retrospective cohort study. BMC Public Health. 2012;12:674.

8. **Hermans SM**, Castelnuovo B, Katabira C, Mbidde P, Lange JM, Hoepelman AI, et al. Integration of HIV and TB services results in improved TB treatment outcomes and earlier prioritized ART initiation in a large urban HIV clinic in Uganda. Journal of Acquired Immune Deficiency Syndromes: JAIDS. 2012;60(2):e29-35.

9. **Huerga** H, Spillane H, Guerrero W, Odongo A, Varaine F. Impact of introducing human immunodeficiency virus testing, treatment and care in a tuberculosis clinic in rural Kenya. International Journal of Tuberculosis & Lung Disease. 2010;14(5):611-5.

10. **Ikeda** JM, Tellez CA, Hudes ES, Page K, Evans J, Racancoj O, et al. Impact of integrating HIV and TB care and treatment in a regional tuberculosis hospital in rural Guatemala. AIDS & Behavior. 2014;18 Suppl 1:S96-103.

11. **Kanara** N, Cain KP, Laserson KF, Vannarith C, Sameourn K, Samnang K, et al. Using program evaluation to improve the performance of a TB-HIV project in Banteay Meanchey, Cambodia. International Journal of Tuberculosis & Lung Disease. 2008;12(3 Suppl 1):44-50.

12. **Kaplan** R, Caldwell J, Hermans S, Adriaanse S, Mtwisha L, Bekker LG, et al. An integrated community TB-HIV adherence model provides an alternative to DOT for tuberculosis patients in Cape Town. International Journal of Tuberculosis & Lung Disease. 2016;20(9):1185-91.

13. **Kerschberger** B, Hilderbrand K, Boulle AM, Coetzee D, Goemaere E, De Azevedo V, et al. The effect of complete integration of HIV and TB services on time to initiation of antiretroviral therapy: a before-after study. PLoS One. 2012;7(10):e46988. Epub 2012/10/17. doi: 10.1371/journal.pone.0046988.

14. **Kufa** T, Fielding KL, Hippner P, Kielmann K, Vassall A, Churchyard GJ, et al. An intervention to optimise the delivery of integrated tuberculosis and HIV services at primary care clinics: results of the MERGE cluster randomised trial. Contemporary Clinical Trials. 2018;72:43-52.

15. **Louwagie** G, Girdler-Brown B, Odendaal R, Rossouw T, Johnson S, Van der Walt M. Missed opportunities for accessing HIV care among Tshwane tuberculosis patients under different models of care. International Journal of Tuberculosis & Lung Disease. 2012;16(8):1052-8.

16. **Mathebula** U, Emerson C, Agizew T, Pals S, Boyd R, Mathoma A, et al. Improving sputum collection processes to increase tuberculosis case finding among HIV-positive persons in Botswana. Public Health Action. 2020;10(1):11-6..

17. **Mwinga** A MN, Kanene C, et al. . Provider-initiated HIV testing and counseling of TB patients--Livingstone District, Zambia, September 2004-December 2006. Mmwr. 2008;Morbidity and mortality weekly report. 57(11):285-9.

18. **Nateniyom** S, Jittimanee SX, Viriyakitjar D, Jittimanee S, Keophaithool S, Varma JK. Provider-initiated diagnostic HIV counselling and testing in tuberculosis clinics in Thailand. International Journal of Tuberculosis & Lung Disease. 2008;12(8):955-61.

19. **Ogarkov** OB, Ebers A, Zhdanova S, Moiseeva E, Koshcheyev ME, Zorkaltseva E, et al. Administrative interventions associated with increased initiation on antiretroviral therapy in Irkutsk, Siberia. Public Health in Action. 2016;6(4):252-4.

20. **Owiti** P, Zachariah R, Bissell K, Kumar AM, Diero L, Carter EJ, et al. Integrating tuberculosis and HIV services in rural Kenya: uptake and outcomes. Public Health in Action. 2015;5(1):36-44.

21. **Rocha** C, Montoya R, Zevallos K, Curatola A, Ynga W, Franco J, et al. The Innovative Socio-economic Interventions Against Tuberculosis (ISIAT) project: an operational assessment. International Journal of Tuberculosis & Lung Disease. 2011;15 Suppl 2:50-7.

22. **Van Rie** A, Patel MR, Nana M, Vanden Driessche K, Tabala M, Yotebieng M, et al. Integration and task shifting for TB/HIV care and treatment in highly resource-scarce settings: one size may not fit all. Journal of Acquired Immune Deficiency Syndromes: JAIDS. 2014;65(3):e110-7.

23. **Van Rie** A, Sabue M, Jarrett N, Westreich D, Behets F, Kokolomani J, et al. Counseling and testing TB patients for HIV: evaluation of three implementation models in Kinshasa, Congo. International Journal of Tuberculosis & Lung Disease. 2008;12(3 Suppl 1):73-8.
